# A short-term mouse model that reproduces the immunopathological features of rhinovirus-induced exacerbation of COPD

**DOI:** 10.1042/CS20140654

**Published:** 2015-05-08

**Authors:** Aran Singanayagam, Nicholas Glanville, Ross P. Walton, Julia Aniscenko, Rebecca M. Pearson, James W. Pinkerton, Jay C. Horvat, Philip M. Hansbro, Nathan W. Bartlett, Sebastian L. Johnston

**Affiliations:** *Airway Disease Infection Section, National Heart and Lung Institute, Imperial College London, London W2 1PG, U.K.; †Priority Research Centre for Asthma and Respiratory Disease, Hunter Medical Research Institute and University of Newcastle, Newcastle, NSW 2305, Australia

**Keywords:** chronic obstructive pulmonary disease, exacerbation, inflammation, mouse models, rhinovirus, AHR, airway hyper-responsiveness, BAL, bronchoalveolar lavage, CCL5, chemokine (C-C motif) ligand 5, COPD, chronic obstructive pulmonary disease, CXCL10, C-X-C motif chemokine 10, FRC, functional residual capacity, H&E, haematoxylin and eosin, IFN, interferon, IL, interleukin, IP-10, interferon γ-induced protein 10, LPS, lipopolysaccharide, MIP-2, macrophage inflammatory protein 2, MPO, myeloperoxidase, PAS, periodic acid–Schiff, PFA, paraformaldehyde, RANTES, regulated on activation, normal T-cell expressed and secreted, RV, rhinovirus, TLC, total lung capacity, TNF, tumour necrosis factor

## Abstract

Viral exacerbations of chronic obstructive pulmonary disease (COPD), commonly caused by rhinovirus (RV) infections, are poorly controlled by current therapies. This is due to a lack of understanding of the underlying immunopathological mechanisms. Human studies have identified a number of key immune responses that are associated with RV-induced exacerbations including neutrophilic inflammation, expression of inflammatory cytokines and deficiencies in innate anti-viral interferon. Animal models of COPD exacerbation are required to determine the contribution of these responses to disease pathogenesis. We aimed to develop a short-term mouse model that reproduced the hallmark features of RV-induced exacerbation of COPD. Evaluation of complex protocols involving multiple dose elastase and lipopolysaccharide (LPS) administration combined with RV1B infection showed suppression rather than enhancement of inflammatory parameters compared with control mice infected with RV1B alone. Therefore, these approaches did not accurately model the enhanced inflammation associated with RV infection in patients with COPD compared with healthy subjects. In contrast, a single elastase treatment followed by RV infection led to heightened airway neutrophilic and lymphocytic inflammation, increased expression of tumour necrosis factor (TNF)-α, C-X-C motif chemokine 10 (CXCL10)/IP-10 (interferon γ-induced protein 10) and CCL5 [chemokine (C-C motif) ligand 5]/RANTES (regulated on activation, normal T-cell expressed and secreted), mucus hypersecretion and preliminary evidence for increased airway hyper-responsiveness compared with mice treated with elastase or RV infection alone. In summary, we have developed a new mouse model of RV-induced COPD exacerbation that mimics many of the inflammatory features of human disease. This model, in conjunction with human models of disease, will provide an essential tool for studying disease mechanisms and allow testing of novel therapies with potential to be translated into clinical practice.

## Abstract 2

The present study describes a new short-term mouse model of rhinovirus (RV)-induced exacerbation of COPD (chronic obstructive pulmonary disease) which will facilitate insight into disease mechanisms and could provide a more efficient tool to test novel therapies with potential to be translated into clinical practice.

## INTRODUCTION

Chronic obstructive pulmonary disease (COPD) is the most common chronic respiratory condition in adults and its prevalence is expected to increase markedly in the future [1]. The clinical course of COPD is punctuated by acute exacerbations, which are a major cause of morbidity and mortality and place a significant burden on healthcare services [2–4]. A number of different agents have been suggested to trigger COPD exacerbations, with respiratory viruses [most commonly rhinoviruses (RVs)] frequently identified [5,6].

Inflammatory responses in the airways during virus-induced exacerbations of COPD are poorly understood. Some insight has been gained from naturally occurring COPD exacerbation studies, but these studies are limited by variability in factors such as time between virus infection and presentation and treatments initiated prior to sampling. To address these issues, we have developed a model of experimental RV-induced COPD exacerbation in humans that allows sequential measurement of a range of clinical and inflammatory parameters and has provided a clearer understanding of the relationship between virus infection, inflammatory responses and biological and physiological markers [7]. Key features of exacerbation in comparison with stable-state COPD reported in this and other human studies include increased neutrophilic [7–12] and lymphocytic [7,9,11,12] cellular airways inflammation, enhanced production of cytokines such as tumour necrosis factor (TNF)-α [7,13], CXCL10 (C-X-C motif chemokine 10)/IP-10 (interferon γ-induced protein 10) [14] and CCL5 [chemokine (C-C motif) ligand 5]/RANTES (regulated on activation, normal T-cell expressed and secreted) [9,10] in the airways, deficient type I interferon responses to RV infection, increased virus load and enhanced airway mucus production [7]. Additionally, RV infection in patients with COPD has been shown to be associated with enhanced airway neutrophilia and lymphocytosis and increased neutrophil chemokine CXCL8/IL-8 expression compared with RV infection in healthy smokers [7,15,16].

Animal models of chronic respiratory diseases have historically played important roles in broadening our understanding of disease mechanisms, including development of the proteinase/anti-proteinase imbalance hypothesis in COPD [17]. A mouse model of RV-induced COPD exacerbation that can mimic what is known of human disease could therefore provide further critical insight into disease mechanisms and be used to test novel therapies. However, this presents a considerable challenge due to a limited understanding of the mechanisms driving underlying COPD and of the distinct clinical phenotypes in humans.

Previously described animal models of COPD have used one of three main approaches: inhalation of noxious stimuli (most commonly cigarette smoke), instillation of tissue-degrading proteinases such as elastase or genetic manipulation [18,28,52]. Cigarette smoke administration models require at least 2 months’ exposure before some of the pathological features of COPD are evident [18]. Models that use instillation of elastase produce a rapid onset of emphysematous destruction of the lungs with mucin induction and may be considered the best short-term method for modelling severe disease. A number of studies have described elastase-induced models of COPD with exacerbation precipitated by bacteria and, more recently, RV infection [19–21]. These models have used various protocols, including single [19,20] or multiple [21,22] doses of intranasal elastase, differing intervals between elastase dosing and infection [19,20,23] and the addition of lipopolysaccharide (LPS) to model chronic bacterial colonization [21,22]. Given this array of approaches, the optimal protocol for recreating the features of virus-induced COPD exacerbation that have been identified in humans is unclear.

In the present study, we describe a 10 day mouse model consisting of a single dose of elastase administration to establish severe emphysematous lung disease followed by RV infection that recreates many of the inflammatory features of human RV-induced COPD exacerbation.

## MATERIALS AND METHODS

### Animals

All studies were performed in 8–10-week-old, wild-type, female C57BL/6 mice, purchased from Charles River Laboratories and housed in individually ventilated cages under specific pathogen-free conditions. During all experiments, animal welfare was monitored at least twice daily.

### COPD models

Isofluorane-anaesthetized mice were intranasally challenged with 1.2 units of porcine pancreatic elastase (Merck) on day 1 and with 70 endotoxin units of LPS from *Escherichia coli* 026:B6 (Sigma–Aldrich) on day 4 of the week for up to 4 consecutive weeks, as previously described [21]. In some experiments, mice were alternatively treated with a single dose of 1.2 units of elastase alone. Mice treated with intranasal PBS instead of elastase or LPS were used as controls.

### RV infection

RV serotype 1B was obtained from the A.T.C.C. and propagated in Ohio HeLa cells, as described previously [24]. Mice were infected intranasally under light isofluorane anaesthesia with 2.5 × 10^6^ tissue culture infectious dose (TCID_50_) RV1B or UV-inactivated RV control either 7 days after final LPS challenge in the case of combined elastase and LPS models or 10 days after elastase challenge in the single-dose elastase model.

### Cytospin assay

Bronchoalveolar lavage (BAL) was performed as previously described [24]. Cells were pelleted by centrifugation, resuspended in ammonium-chloride-potassium (ACK) buffer to lyse red blood cells, washed with PBS and resuspended in RPMI 1640 medium with 10% FBS. Cells were then spun on to slides and stained with Quik-Diff (Reagena) for differential counts. Counts were performed blinded to experimental conditions.

### ELISA

Cytokine and chemokine protein levels in BAL were measured using commercial duoset ELISA kits (R&D Systems), according to the manufacturer's instructions.

### Myeloperoxidase assay

To indirectly assess neutrophil activation, the chlorination activity of released myeloperoxidase (MPO) was measured in BAL using the EnzChek MPO activity assay kit (Invitrogen), according to the manufacturer's instructions.

### Quantitative real-time PCR

Total RNA was extracted from the excised apical lobe of the right mouse lung using an RNeasy mini kit (Qiagen) and reverse transcribed using 5 μg of random hexamers (Omniscript RT kit, Qiagen) as primers. Quantitative PCR was carried out using specific primers and probes for each gene and normalized to 18S rRNA. Primers and probes for *18S* [24], RV [25], interferon β (*IFN-β*) [25], interferon λ (*IFN-λ*) [25], *MUC5A*C [24] and *MUC5B* [24] have been described previously. The following primer and probe sequences were used for interleukin (*IL*)*-13* and *TNF-α*: IL-13 forward 5'-GATATTGCATGGCCTCTGTAACC-3′, IL-13 reverse 5′-GGGCTACTTCGATTTTGGTATCG-3′, IL-13 probe 5′-FAM-CAAGGCCCCCACTACGGTCTCCA-TAMRA;-3′ TNF-α forward 5′-CATCTTCTCAAAATTCGAGTGACAA-3′, TNF-α reverse 5′-TGGGAGTAGACAAGGTACAACCC-3′, TNF-α probe 5′-FAM-CACGTCGTAGCAAAC-TAMRA-3′.

### Histopathological analysis

Following BAL, lungs were perfused with PBS via the heart and inflated with 4% paraformaldehyde (PFA), then immersion fixed in 4% PFA for 24 h. Fixed lung samples were embedded in paraffin wax and 5-μm-thick histological sections were cut and stained with haematoxylin and eosin (H&E) or periodic acid–Schiff (PAS). Mean linear intercept was determined by measuring the diameter of air spaces in ten random fields per slide using Zeiss Axiovision software v4.8.3.0. PAS staining was scored using a system described previously [26]. Ten to twenty airways were counted per section. All counting was performed blind to experimental conditions.

### Assessment of lung function

Lung function was assessed as previously described [18]. Mice were anaesthetized with ketamine (125 mg/kg) and xylazine (16 mg/kg) and were then cannulated (tracheostomy with ligation). Work of breathing, functional residual capacity (FRC), total lung capacity (TLC) and dynamic lung compliance were measured using a forced pulmonary manoeuvre system (Buxco). An average breathing frequency of 200 breaths/minute was applied to anaesthetized animals. Each manoeuvre was performed a minimum of three times and the average was calculated. Dynamic compliance readings were taken every 2 s for 2 min and the average was calculated. The FlexiVent FX1 apparatus (SCIREQ) was used to assess hysteresis and tissue damping. Maximal pressure/volume (PV) loops were used to calculate hysteresis. For all perturbations, a coefficient of determination of 0.95 was the minimum allowable for an acceptable measurement. Each perturbation was conducted three times per animal and the average was calculated, with a minimum ventilation period of 20 s allowed between each perturbation.

### Assessment of airways hyper-responsiveness

Airway hyper-responsiveness (AHR) was measured as enhanced pause (PenH) in response to nebulized challenge with methacholine, using an unrestrained whole-body plethysmography system (Electromedsystems), as previously described [26]. PenH is displayed as average values for a 5 min log period post-methacholine challenge.

### Statistical analyses

Mice were studied in groups of four or five and data are presented as means±S.E.M., representative of or comprising at least two independent experiments. Data were analysed by ANOVA and Bonferroni's multiple comparison test. All statistics were calculated using Prism 4.2 software (GraPhPad).

### Study approval

All animal work was completed in accordance with U.K. Home Office guidelines (U.K. project licence PPL 70/7234).

## RESULTS

### Multiple doses of elastase and LPS in combination with RV infection do not accurately model COPD exacerbation

We initially attempted to reproduce a previously reported mouse model of RV-induced COPD exacerbation [21] using exactly the same dosing protocol of once weekly intranasal elastase and LPS administration for 4 weeks followed by RV infection (Supplementary Figure S1a). Consistent with the previous report of this model, we found that induction of *IFN-β* and *IFN-λ* mRNAs in lung tissue *in vivo* were reduced with 4 weeks of elastase/LPS administration followed by RV infection (elastase/LPS + RV) compared with treatment with PBS and infection with RV (PBS+RV; modelling RV infected healthy subjects) (Figures S1b and S1c). Lung tissue *IL-13* mRNA was also increased in elastase/LPS + RV-treated mice compared with either treatment alone (Figure S1d), as previously reported [21].

However, in contrast with the original report of this model, we found that elastase/LPS treatment followed by RV infection led to reduced rather than increased lung virus loads compared with non-COPD mice infected with RV (Figure S1e), reduced rather than increased expression of *TNF-α* and no difference in *MUC5AC* mRNA levels in lung tissue compared with PBS + RV-treated mice (Supplementary Figures S1f and S1g). AHR was increased in mice treated with elastase/LPS + RV compared with PBS + RV but was reduced compared with mice treated with elastase/LPS + UV (Supplementary Figure S1h).

We also measured a number of other inflammatory endpoints associated with human disease that were not originally reported [21]. BAL neutrophil numbers on day 1 and BAL lymphocyte numbers on day 4 post-challenge were increased with RV infection as previously shown [24] (PBS + RV compared with PBS + UV, Supplementary Figures S2a and S2b). BAL neutrophil numbers were increased in elastase/LPS + RV-compared with elastase/LPS + UV-treated mice, but were decreased compared with PBS + RV treatment at day 1 post-challenge (Supplementary Figure S2a). BAL lymphocyte numbers were no different in elastase/LPS + RV-compared with elastase/LPS + UV-treated mice, but were increased on day 1 post-challenge compared with PBS + RV treatment (Supplementary Figure S2b). Levels of the virus-inducible chemokines CXCL10/IP-10, CCL5/RANTES and CXCL2/macrophage inflammatory protein 2 (MIP-2) in BAL were increased by RV infection compared with uninfected controls (PBS + RV compared with PBS + UV treatment), but were not increased in elastase/LPS + RV-compared with PBS + RV-treated mice and CXCL10/IP-10 was reduced in elastase/LPS + RV compared with PBS + RV administration at day 4 post-challenge (Supplementary Figures S2c–S2e). MUC5AC protein levels in BAL on day 4 post-challenge were increased with RV infection alone (PBS + RV compared with PBS + UV; Supplementary Figure S2f) and were also increased in elastase/LPS + RV- compared with PBS + RV-treated mice on day 1 post-infection, but significantly decreased compared with elastase/LPS + UV treatment at the same time point (Supplementary Figure S2f).

### Comparison of single-compared with multiple-dose elastase and LPS to model COPD

**Figure 1:**
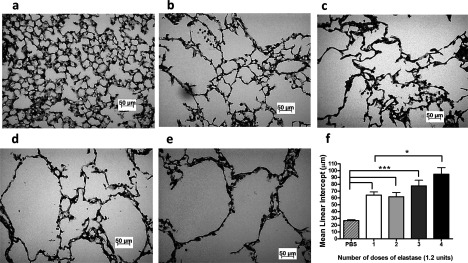
Single elastase/LPS treatment is sufficient to induce emphysema Mice were challenged intranasally with elastase on day 1 and LPS on day 4 of each week or PBS as control for 1, 2, 3 or 4 weeks. At day 7, following final LPS or PBS challenge, lungs were formalin-fixed, paraffin-embedded and stained with H&E. Representative images of mice treated with (**a**) PBS, (**b**) single dose of elastase and LPS, (**c**) two doses of elastase and LPS, (**d**) three doses of elastase and LPS, and (**e**) four doses of elastase and LPS. Scale bars: 50 μm. Magnification ×100 (**f**) The diameter of air spaces were measured in at least ten random fields per slide and were averaged to determine mean linear intercept. *n*=4 mice/group. Data were analysed by ANOVA and Bonferroni post-hoc test. *P<0.05; ***P<0.001.

In our hands, RV infection in the 4-week elastase/LPS COPD model failed to produce most of the inflammatory features of human COPD exacerbation. We speculated that inducing very severe lung damage with multiple doses of elastase interfered with virus infection and associated inflammatory responses, as previously reported [19]. We therefore investigated whether reducing the number of doses of elastase/LPS could still induce significant alveolar destruction with less severe lung damage. Initial comparisons of one, two, three and four weekly doses of elastase and LPS indicated a dose-dependent increase in emphysematous lung changes apparent both visually in H&E-stained lung sections (Figures 1a–1e) and when quantified by measuring mean linear intercept (Figure 1f). A single dose of elastase and LPS was sufficient to induce emphysematous lung changes as defined by significantly increased mean linear intercept compared with control PBS-treated mice (Figure 1f). Despite the histological changes induced by intranasal elastase with or without LPS administration, none of the animals studied showed any outward signs of illness or respiratory compromise, regardless of the dosing protocol used.

**Figure 2:**
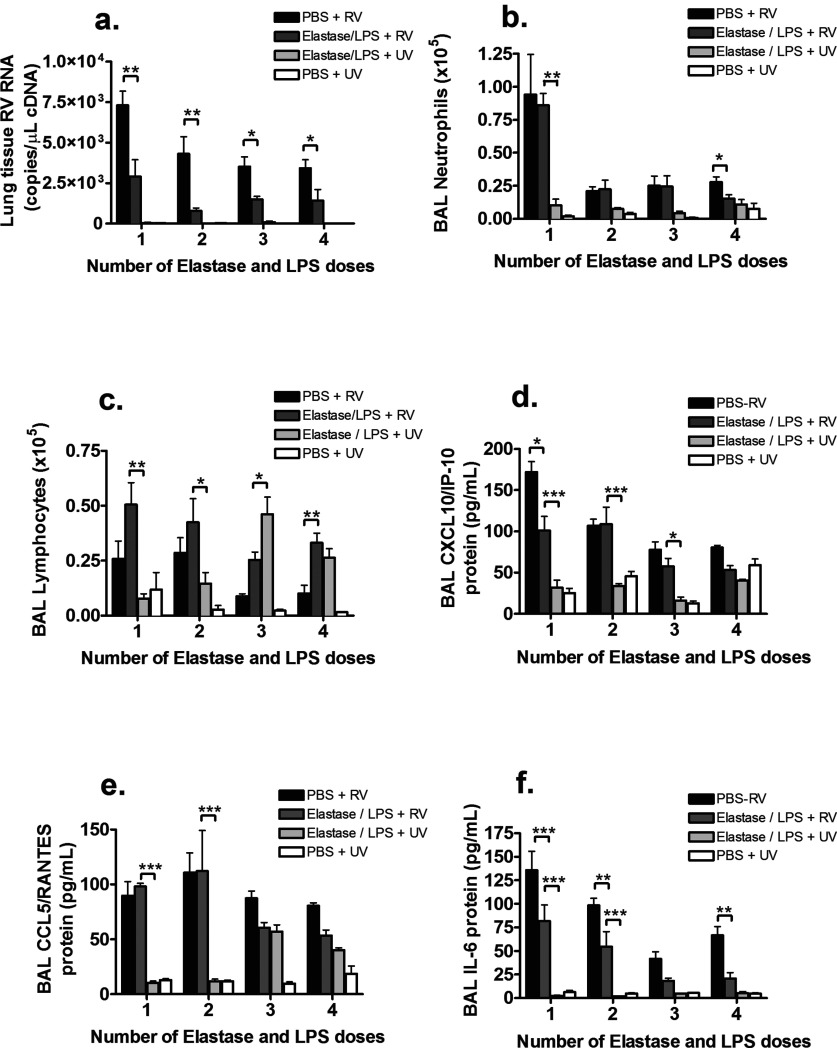
Effect of differing elastase and LPS dosing protocols on RV load and RV-induced airway inflammation Mice were challenged intranasally with elastase on day 1 and LPS on day 4 of each week or PBS as control for 1, 2, 3 or 4 weeks. At day 7 following final LPS or PBS challenge, mice were additionally challenged with RV1B or UV-inactivated RV1B 7 days after final LPS challenge. (**a**) RV RNA copies in lung tissue were measured by Taqman quantitative PCR at 24 h post-infection. (**b**) Neutrophil numbers at 24 h post-infection and (**c**) lymphocyte numbers at day 4 post-infection were enumerated in BAL by cytospin assay. (**d**) CCL5/RANTES, (**e**) CXCL10/IP-10 and (**f**) IL-6 proteins at 24 h post-infection were measured in BAL by ELISA. *n*=5 mice/group. Data were analysed by two-way ANOVA and Bonferroni post-hoc test. *P<0.05; **P<0.01; ***P<0.001.

To determine whether reducing elastase/LPS-induced lung damage increased responses to infection, we compared single with up to four doses of elastase and LPS followed by RV infection. Regardless of the number of doses administered, elastase/LPS failed to enhance RV-induced airway inflammation. We observed reduced viral RNA levels in lung tissue (Figure 2a) and reduced or no difference in BAL neutrophilia, BAL lymphocytosis (except for the four-dose protocol) and BAL CXCL10/IP-10, CCL5/RANTES and IL-6 in elastase/LPS + RV-compared with PBS + RV-treated mice (Figures 2d–2f). The number of doses of elastase and LPS therefore had little effect on the efficacy of this model when comparing elastase/LPS + RV treatment to RV infection alone. However, a number of inflammatory endpoints including BAL neutrophilia (one-dose elastase/LPS protocol), BAL lymphocytosis (one- and two-dose protocols) and protein levels of CXCL10/IP-10 (one-, two- and three-dose protocols), CCL5/RANTES (one- and two-dose protocols) and IL-6 (one-, two- and four-dose protocols) in BAL were increased in elastase/LPS + RV- treated mice compared with elastase/LPS + UV-treated mice (Figures 2b–2f).

### Single-dose elastase in combination with RV infection more accurately models COPD exacerbation

**Figure 3:**
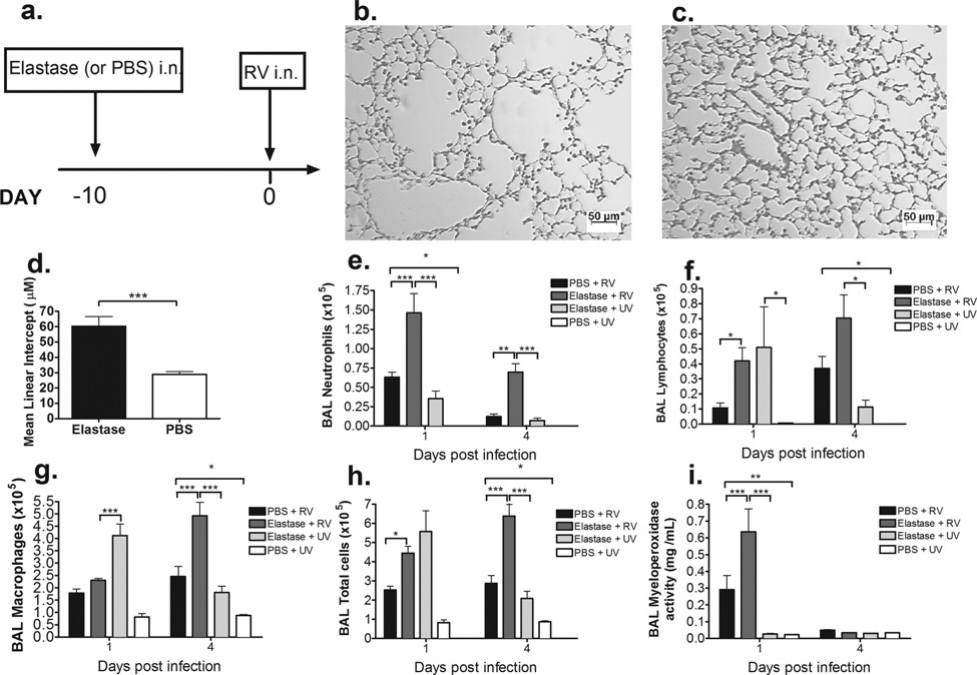
Single-dose elastase treatment induces histological emphysema and enhances pulmonary inflammation in RV-infected mice (**a**) Mice were challenged intranasally with a single dose of elastase or PBS as control and at day 10 post-challenge, lungs were formalin-fixed, paraffin-embedded and stained with H&E. Representative images of mice treated with (**b**) elastase and (**c**) PBS. Scale bars: 50μm. Magnification ×100 (**d**). The diameter of air spaces were measured in at least ten random fields per slide and averaged to determine mean linear intercept. On day 10 after elastase or PBS challenge, mice were additionally challenged intranasally with RV1B or UV-inactivated RV1B (UV). (**e**) Neutrophil, (**f**) lymphocyte, (**g**) macrophage and (**h**) total cell numbers in BAL were enumerated by cytospin assay. (**i**) MPO activity was measured indirectly by assessment of chlorination of 3′-(*p*-aminophenyl fluorescein) in BAL. *n*=5 mice/group. Data were analysed by two-way ANOVA and Bonferroni post-hoc test. *P<0.05; **P<0.01; ***P<0.001.

Alternative mouse models of COPD have successfully used single-dose elastase administration protocols and demonstrated enhanced inflammatory responses to bacterial challenge [19,20]. Since the combination of elastase and LPS with RV did not produce a phenotype that we considered to be consistent with human COPD exacerbation, regardless of the number of doses administered, we reasoned that LPS may be activating innate immunity and thus directly interfering with RV infection. We therefore determined whether removal of the LPS component from the protocol would lead to a more representative disease model (Figure 3a). Similarly to combined elastase/LPS, single-dose elastase administration to mice caused histological emphysematous lung changes compared with PBS-treated controls (Figures 3b–3d). Numbers of neutrophils (days 1 and 4 post-challenge) and MPO activity (day 1 post-challenge) in BAL were both significantly greater in elastase + RV-treated mice compared with either elastase administration or RV infection alone (Figures 3e and 3i). Lymphocytes in BAL were greater on day 1 post-challenge in mice treated with elastase + RV compared with PBS + RV treatment and on day 4 post-challenge compared with elastase + UV treatment (Figure 3f). Total cell and macrophage numbers in BAL were increased in elastase + RV-compared with both elastase + UV-and PBS + RV-treated mice at day 4 post-challenge (Figures 3g and 3h).

**Figure 4:**
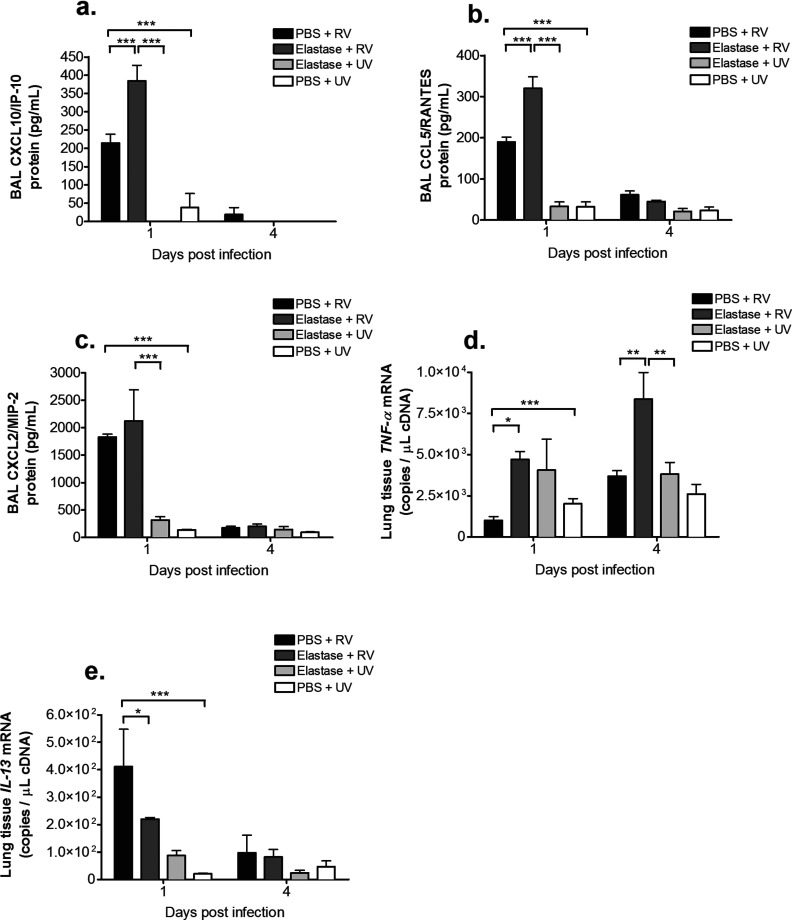
Single-dose elastase treatment enhances inflammatory chemokine and cytokine production in RV-infected mice (**a**) Mice were challenged intranasally with a single dose of elastase or PBS as control. On day 10 after elastase or PBS challenge, mice were additionally challenged intranasally with RV1B or UV-inactivated RV1B (UV). (**a**) CXCL10/IP-10, (**b**) CCL5/RANTES and (**c**) CXCL2/MIP-2 proteins were measured in BAL by ELISA. (**d**) *TNF-α* and (**e**) *IL-13* mRNA in lung tissue was measured by Taqman quantitative PCR. *n*=5 mice/group. Data were analysed by two-way ANOVA and Bonferroni post-hoc test. *P<0.05; **P<0.01; ***P<0.001.

We also observed significant increases in BAL protein levels of CXCL10/IP-10 and CCL5/RANTES (day 1 post-challenge) and lung tissue *TNF-α* mRNA expression (day 4 post-challenge) in elastase + RV-treated mice compared with either PBS + RV or elastase + UV treatments (Figures 4a, 4b and 4d). In addition, BAL protein levels of CXCL2/MIP-2 were increased in elastase + RV-compared with elastase + UV-treated mice at day 1 post-challenge (Figure 4c). Lung tissue gene expression of *IL-13* was significantly lower in elastase + RV- compared with PBS + RV-treated mice (Figure 4e).

**Figure 5:**
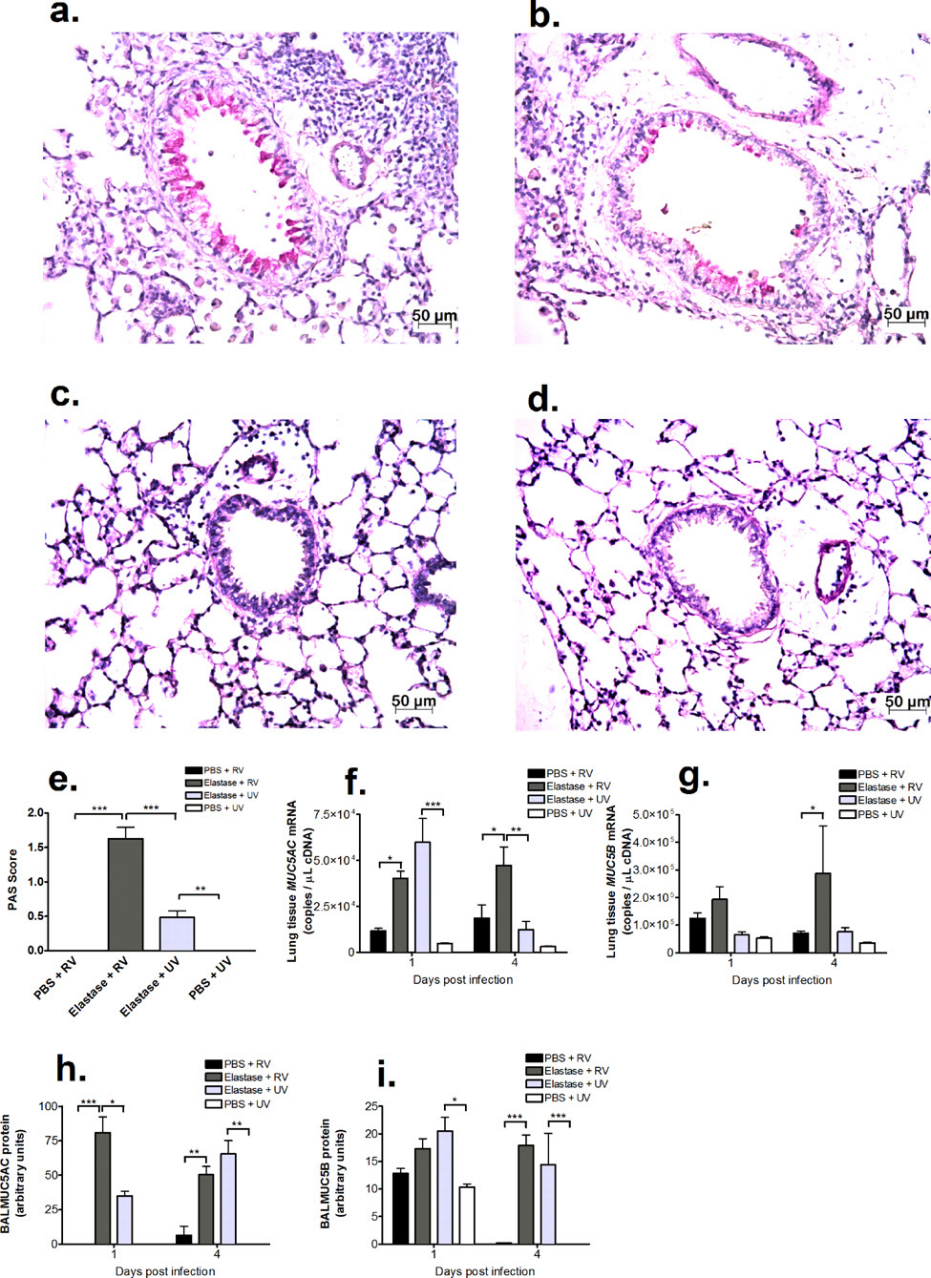
RV infection enhances mucus production in a single-dose elastase COPD model Mice were challenged intranasally with a single dose of elastase or PBS as control. Ten days later, mice were infected intranasally with RV1B or UV-inactivated RV1B (UV). At day 4 after RV challenge, lungs were formalin-fixed, paraffin-embedded and stained with PAS. Representative images of mice treated with (**a**) elastase + RV1B, (**b**) elastase + UV, (**c**) PBS + RV1B and (**d**) PBS + UV. Scale bars: 50 μm. Magnification ×400 (**e**) Scoring for PAS-positive mucus-producing cells. (**f**) *MUC5AC* and (**g**) *MUC5B* mRNA in lung tissue was measured by Taqman quantitative PCR. (**h**) MUC5AC and (**i**) MUC5B proteins were measured in BAL by ELISA. *n*=5 mice/group. Data were analysed by two-way ANOVA and Bonferroni post-hoc test. *P<0.05; **P<0.01; ***P<0.001.

Increased mucus production and mucus plugging of the airways is a recognized feature of COPD and has been shown to be further increased by RV infection [27]. Staining of lung sections with PAS revealed abundant PAS-positive mucus-producing cells in the airways of elastase + RV-treated mice 4 days after RV challenge and, to a significantly lesser extent, in the airways of elastase + UV-treated mice (Figures 5a, 5b and 5e). No PAS-positive cells were visible in the airways of mice receiving PBS in combination with either RV or UV-inactivated virus (Figures 5c, 5d and 5e). We also assessed airway mucin gene and protein levels. On day 4, after virus infection lung tissue *MUC5AC* mRNA levels were increased in elastase + RV- compared with PBS + RV- and elastase + UV-treated mice (Figure 5f). Lung tissue *MUC5AC* mRNA levels were similarly increased compared with PBS + RV treatment, but not compared with elastase + UV treatment, at day 1 (Figure 5f). Lung *MUC5B* mRNA levels were increased at day 4 post-challenge in elastase + RV- compared with PBS + RV-treated mice (Figure 5g). BAL MUC5AC protein levels were also increased in elastase + RV- compared with PBS + RV-treated mice at both time-points and compared with elastase + UV-treated mice at day 1 post-challenge (Figure 5h). BAL MUC5B protein was increased in elastase + RV- compared with PBS + RV-treated mice on day 4 post-challenge (Figure 5i).

**Figure 6:**
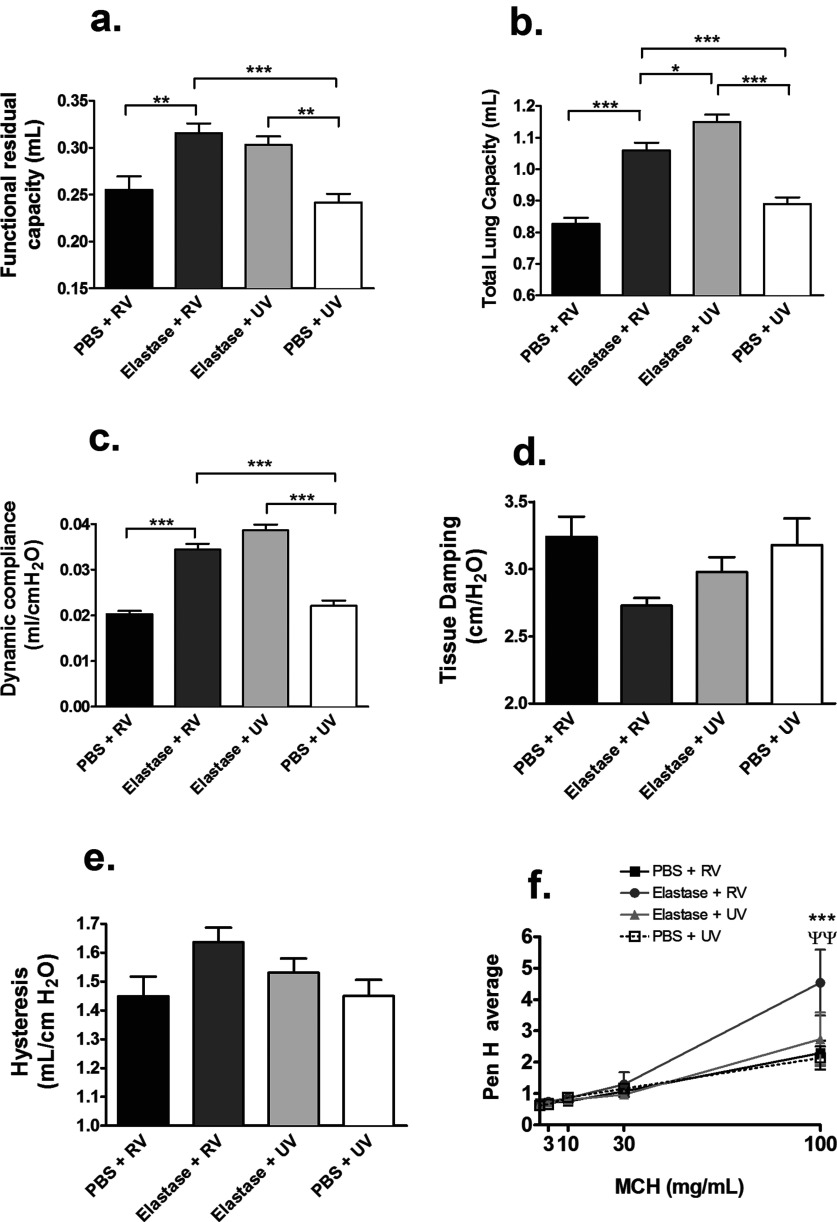
Single-dose elastase treatment induces lung function changes Mice were challenged intranasally with a single dose of elastase or PBS as control. Ten days later, mice were infected intranasally with RV1B or UV-inactivated RV1B (UV). At day 1 after RV challenge, forced manoeuvre techniques and Flexivent were used to assess lung function parameters including (**a**) FRC, (**b**) TLC, (**c**) dynamic compliance, (**d**) tissue damping and (**e**) lung hysteresis. (**f**) AHR was measured by whole-body plethysmography at day 1 post-infection. (**a**–**e**) *n*=10 mice/group, two independent experiments combined. Data analysis by one-way ANOVA and Bonferroni post-hoc test (**f**) *n*=8 mice/group, two independent experiments combined. Data analysis by two-way ANOVA and Bonferroni post-hoc test. *P<0.05; **/ψψ*P*<0.01; ***P<0.001. In (**f**), * indicates statistical comparison between elastase + RV and PBS + RV groups and ψ indicates comparison between elastase + RV and elastase + UV groups.

Assessment of lung function parameters in the single-dose elastase model showed abnormalities consistent with human COPD including increased FRC, TLC and increased dynamic lung compliance associated with elastase administration (elastase + UV compared with PBS + UV-treated mice; Figures 6a–6c). We did not observe any additional effect of RV infection on these abnormal parameters at day 1 post-challenge with no increases in FRC, TLC or dynamic compliance observed in elastase + RV- compared with elastase + UV-treated mice (Figures 6a–6c). There were no significant effects of elastase treatment and/or RV infection on tissue damping or lung hysteresis (Figures 6d and 6e). We also assessed AHR measured as PenH using whole-body plesmythography at 24 h post-RV challenge. Neither RV infection nor elastase treatment alone caused increased AHR compared with PBS + UV-treated controls. However, mice exposed to single-dose elastase followed by RV infection had significantly increased PenH at the highest dose of methacholine compared with PBS + RV- or elastase + UV-treated mice (Figure 6f).

**Figure 7:**
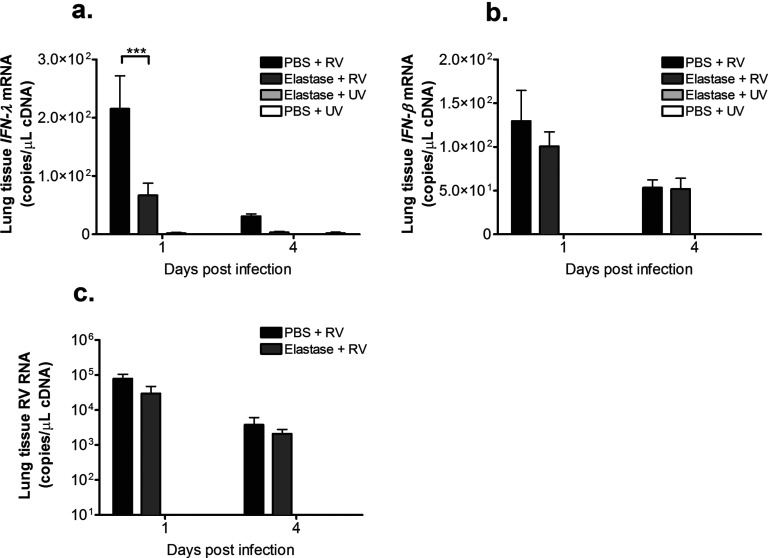
Deficient IFN-λ production in RV-infected mice with elastase-induced COPD Mice were challenged intranasally with single-dose elastase or PBS as control. On day 10 after elastase or PBS challenge, mice were additionally challenged intranasally with RV1B or UV-inactivated RV1B (UV). (**a**) *IFN-λ* mRNA, (**b**) *IFN-β* mRNA and (**c**) RV RNA in lung tissue was measured by Taqman quantitative PCR. *n*=5 mice/group. Data were analysed by two-way ANOVA and Bonferroni post-hoc test. ***P<0.001.

In our human model of RV-induced COPD exacerbation, there was evidence of a deficiency in type I interferon responses to RV [7]. We therefore assessed innate anti-viral immune responses and virus loads in the single-dose elastase-induced COPD model. Lung tissue IFN-λ levels were reduced in elastase + RV- compared with PBS + RV-treated mice on day 1 post-infection (Figure 7a). There was no significant difference in lung *IFN-β* mRNA levels (Figure 7b) and no significant effect of elastase treatment on lung tissue RV RNA levels on either day 1 or day 4 post-infection (Figure 7c).

## DISCUSSION

Respiratory viral infections, especially with RVs, are associated with a large proportion of COPD exacerbations [6,8], but understanding of the mechanisms by which viral infection enhances disease is severely lacking. The development of mouse models of COPD exacerbation, in parallel with the existing human experimental model [7], will allow insight into disease mechanisms and testing of potential therapies. In the present study, we report a new mouse model of RV-induced COPD exacerbation. Our model is simple in comparison with the other existing animal model of RV-induced COPD exacerbation [21], comprising just a single intranasal administration of porcine pancreatic elastase, followed by infection with minor group RV1B. We found that our model mimics many of the key pathological features reported in human experimental and naturally occurring disease, including enhanced neutrophilic and lymphocytic airways inflammation, exaggerated inflammatory cytokine production and increased airways mucus production.

A variety of mouse models of COPD have previously been described including various transgenic strains (e.g. overexpression of matrix metalloproteinase-1 [28] or IL-13 [29]) and cigarette smoke exposure [18]. Our base model of COPD comprises administration of porcine pancreatic elastase to induce emphysematous lung damage. A criticism of this model is that it does not employ the primary disease-causing agent unlike models based on cigarette smoke administration. However, smoke-exposure models are acknowledged to be complex to set up, require prolonged exposure and do not induce significant emphysematous changes or lung function abnormalities consistent with advanced disease. It is also notable that only 15–20% of smokers develop COPD [30], thereby suggesting that cigarette smoke exposure alone is insufficient to generate disease. Additionally, protease dysregulation can also cause COPD in humans (in the case of patients with α-1 anti-trypsin deficiency), thereby providing further rationale for use of elastase to induce features of COPD in mice. Furthermore, acute exacerbations of disease become more frequent as the disease progresses [31] and, therefore, elastase models may be more appropriate when studying pathophysiological mechanisms involved in exacerbations. Some previous studies have combined cigarette smoke exposure with influenza or respiratory syncytial virus infection to model COPD exacerbation in mice [32,33]. These studies have reported various effects of cigarette smoke including increased [33] or reduced [34] virus loads and enhanced [32,33] or suppressed [35] airway inflammation. However, other disease-relevant parameters such as mucus hypersecretion and lung function impairments have not been assessed in these models and, to date, no study has combined cigarette smoke exposure with RV infection in mice.

Airway inflammation is known to be a key underlying pathological process in COPD and neutrophilic inflammation is a recognized characteristic of COPD in both stable-state and during exacerbations [7,36,37]. In our initial efforts to try to reproduce a published model [21] and then to try to optimize this model, we found that multiple doses of elastase and LPS led to suppression or no change rather than enhancement of RV-induced airways neutrophilia and levels of inflammatory cytokines such as TNF-α, CXCL10/IP-10, CCL5/RANTES and CXCL2/MIP-2 compared with control PBS-dosed and RV-infected mice, the equivalent of an RV-infected healthy control patient. This effect on neutrophilia in particular could be due to the LPS component of the model because a previous comparison showed attenuated BAL neutrophilia with chronic compared with acute LPS challenge which was believed to be due to the resolution phase of acute inflammation preventing further neutrophil recruitment [38]. Additionally, a recent *in vitro* study demonstrated that LPS administration attenuates RV-induced neutrophil chemokine expression [39]. More generally, the lack of enhancement of airway inflammation is also in keeping with a previous study in which a very high dose of elastase was administered to mice (12 units, 10-fold higher than in the present study) leading to severe lung damage and impairment of subsequent inflammatory responses to *Streptococcus pneumoniae* [19]. It was speculated that this could be a consequence of airway epithelial damage or perhaps altered alveolar macrophage function [19]. Therefore, given this finding that severe lung damage can suppress the inflammatory response to pathogens and the fact that chronic LPS challenge in itself also causes emphysematous lung damage [38,40], it is perhaps not surprising that chronic challenge with both of these agents led to suppression of inflammatory responses to RV. In contrast, our model of single-dose elastase led to significantly increased neutrophil numbers in the BAL compared with naive mice with further significant increases in neutrophilia at days 1 and 4 post-infection when elastase was combined with RV infection compared with either treatment alone. In addition to increased neutrophil numbers in elastase-treated mice infected with RV, we also observed concomitant increased MPO activation, a protein that is released from primary neutrophil granules following activation [41]. It is known that neutrophil activation markers are increased in sputum of patients with COPD compared with healthy controls [42,43] and previous studies have also reported increased MPO activity in sputum [13] or exhaled breath condensate [44] of patients with COPD during exacerbations.

We also observed increased BAL lymphocytosis in mice receiving single-dose elastase followed by RV compared with control mice treated with PBS and RV or mice treated with elastase and UV-inactivated virus. This finding is also in keeping with our human model of COPD RV exacerbation where increased lymphocytes in BAL were seen at 7 days after RV infection in patients with COPD compared with healthy controls [7] with a predominance of CD8^+^ T-cells [16]. Whether this represents an appropriate or exaggerated response to RV infection and/or contributes to lung parenchymal damage in COPD is unclear [16]. Further consistent with human studies, we observed increases in airway inflammatory cytokines in single-dose elastase and RV-treated mice compared with either treatment alone, including CXCL10/IP-10, CCL5/RANTES and TNF-α which have all been shown to be up-regulated during naturally occurring COPD exacerbations in comparison with stable state [7,9,10,13,45].

Mucus hypersecretion and plugging of the airways is another cardinal feature of COPD and increased MUC5AC and MUC5B production has been demonstrated in histopathological specimens from patients with COPD [46]. Furthermore, RV has been shown to increase airway mucins *in vitro* [27,47] and *in vivo* [24,48], and increased sputum production is a key symptom described during experimental exacerbations of disease [7]. In our model, we found increases in lung tissue gene expression and BAL protein levels of the major respiratory mucins MUC5AC and MUC5B in mice treated with elastase followed by RV compared with control mice receiving PBS followed by RV. There is considerable interest in selective therapeutic targeting of mucin production in COPD and our mouse model provides an *in vivo* system that may facilitate mechanistic dissection of the pathways involved to aid development of therapeutic targets.

Acute exacerbations of COPD are associated with increased airway obstruction, which is believed to be secondary to inflammation and mucus hypersecretion [49]. In our human model of disease, we observed significant reductions in post-bronchodilator peak expiratory flow in patients with COPD infected with RV [7]. Assessment of airway resistance by whole-body plethysmography in our single-dose elastase mouse model did not show any baseline differences between mice treated with elastase compared with mice treated with PBS, but we did observe increased AHR to methacholine challenge in mice exposed to elastase and RV compared with treatment with elastase or infection with RV alone. AHR is considered to be a hallmark feature of asthma, but is increasingly being recognized as a feature in COPD [50]. However, it should be noted that the applicability of non-invasive measurements of lung function such as whole-body plesmythography may be questionable, as the technique does not provide a direct assessment of lung mechanics and thus may not be the optimum method for measuring lung function changes associated with chronic obstructive lung disorders such as COPD. We therefore additionally used invasive techniques to directly measure lung function in our model and found single-dose elastase induced abnormalities consistent with human COPD including increased TLC and FRC and increased pulmonary compliance. Similar findings have been reported in previous studies that have utilized single-dose elastase mouse models of COPD [51,52]. In contrast with whole-body plethysmography, we did not observe additional worsening of these parameters when RV infection was combined with elastase treatment.

Our model of elastase-induced COPD did not, however, recreate all features of human RV-induced COPD exacerbation that have been reported. In our human model of experimental COPD exacerbation we observed that deficient RV induction of IFN-β in stable COPD *ex vivo* was followed by increased virus load following subsequent RV infection *in vivo* [7]. However, all of the experimental protocols we assessed in mice, including single-dose elastase and up to four doses of elastase and LPS, led to similar or lower lung RV RNA levels compared with control PBS + RV-treated mice. These lower virus loads were accompanied by the expected lower levels of IFN-β and IFN-λ in lung tissues taken at the same time points *in vivo*. The lower virus loads and accompanying lower levels in interferon induction *in vivo* might, in part, be explained by the fact that the intranasal elastase mouse model is associated with mucus hypersecretion in the large airways (as shown by PAS-positive staining in the airway lining). This may theoretically impair efficient binding of RV to the bronchial epithelium and thereby lead to a reduction in virus loads, as demonstrated by a previous study which reported reduced virus loads following influenza virus challenge in *MUC5AC*-overexpressing mice [53]. We are unable to explain the difference between our results in mice (lower virus loads accompanied by the expected lower levels of IFN-β and IFN-λ in lung tissues taken at the same time points *in vivo*) and the findings in the previous mouse model study employing four doses of elastase and LPS [21] which reported the surprising findings of greater virus loads accompanied by absent induction of IFN-α and IFN-β in lung tissues taken at the same time point *in vivo*. We also cannot explain the differences between our results reporting deficient RV induction of IFN-β in BAL cells from stable COPD subjects *ex vivo* [7] and work from the same group in air/liquid interface-cultured bronchial cells from patients with moderate-to-severe COPD which demonstrated enhanced virus replication but increased rather than decreased interferon induction at the same time points [54]. There may be subtleties in design that can explain these apparently contradictory findings, but relationships between interferon responses to RV infection and virus replication *in vitro* and *in vivo* in COPD clearly require further study in both humans and mice. It is also notable that, despite type I and III interferon responses being unchanged or reduced in our single-dose elastase + RV model, BAL protein levels of the interferon-stimulated gene CXCL10/IP-10 were actually enhanced. However, RV may induce certain interferon-stimulated genes, independently of type I interferon signalling [55], and other mediators such as TNF-α, which was enhanced in our model, have been shown to up-regulate CXCL10/IP-10 *in vitro* [56]

In summary, we report a mouse model of RV infection in COPD that mimics a number of inflammatory features of human disease. This model, in conjunction with our human model, will provide a useful tool for studying disease mechanisms and will allow testing of novel therapies with potential to be translated into clinical practice.

## CLINICAL PERSPECTIVES

• RV infections commonly trigger exacerbations in patients with COPD and are a major cause of morbidity and mortality. There is a lack of understanding of the underlying immunopathological mechanisms involved in virus-induced exacerbations and no available effective therapies.
• The aim of the present study was to establish a mouse model that reproduces the hallmark features of RV-induced exacerbation of COPD.
• A single elastase treatment followed by RV infection in mice mimicked a number of hallmark inflammatory features of human disease including enhanced cellular airways inflammation, increased inflammatory cytokine expression and mucus hypersecretion. This model will provide a useful tool for studying disease mechanisms and allow future testing of novel therapies with potential to be translated into clinical practice.

## Online data

Supplementary data
