# Supplementary material for: A short-term mouse model that reproduces the immunopathological features of rhinovirus-induced exacerbation of COPD
Source: Clin Sci (Lond). 2015 May 8;129(Pt 3):245–58. doi: 10.1042/CS20140654 (PMC4557402; doi:10.1042/CS20140654)
Supplement: Supplementary data [file cs1290245ntsadd.pdf]

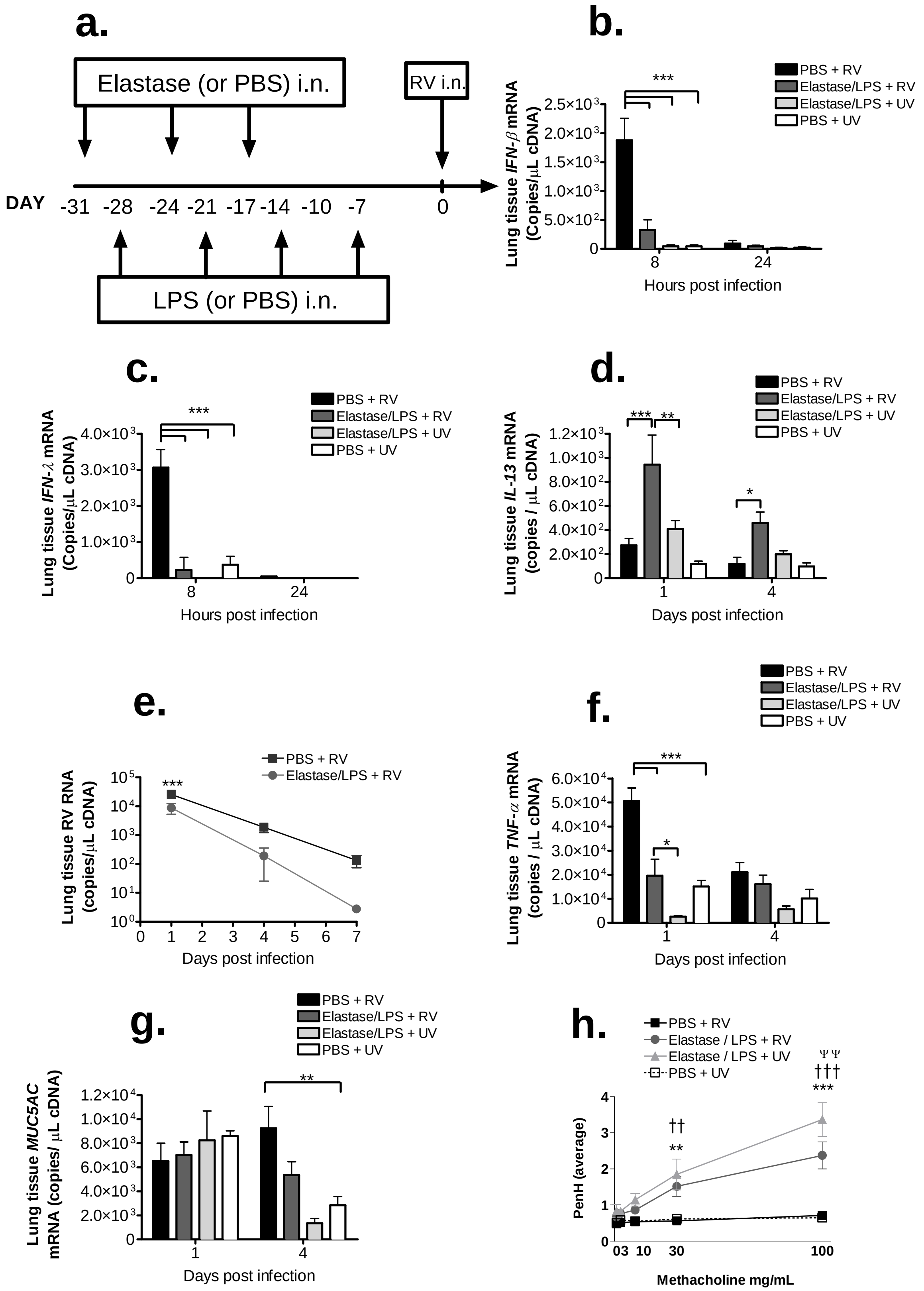

Figure S1: **Attenuated virus-induced responses in four dose elastase/LPS COPD model.**

(a) Mice were challenged intranasally with elastase on day 1 and LPS on day 4 of each week, or challenged with PBS control, for four weeks. At day 7 following final LPS or PBS challenge, mice were additionally challenged with rhinovirus 1B or UV-inactivated RV1B (UV). (b) *IFN-β,* (c) *IFN-λ* mRNA (d) RV RNA, (e) *TNF-α,* (f) *Muc5AC* and (g) *IL-13* mRNA copies in lung tissue were measured by Taqman quantitative PCR. (h) Airway hyperresponsiveness to methacholine was measured by whole body plesmythography at 24 hours post infection. n=5 mice/group. Data were analysed by two-way ANOVA and Bonferroni post-test. *p<0.05; **<0.01; ***p<0.001. In (h) * denotes statistical comparison between elastase/LPS + RV and PBS + RV groups, ł denotes comparison between elastase/LPS + UV and PBS + UV groups, ψ denotes comparison between elastase/LPS + RV and elastase/LPS + UV groups.

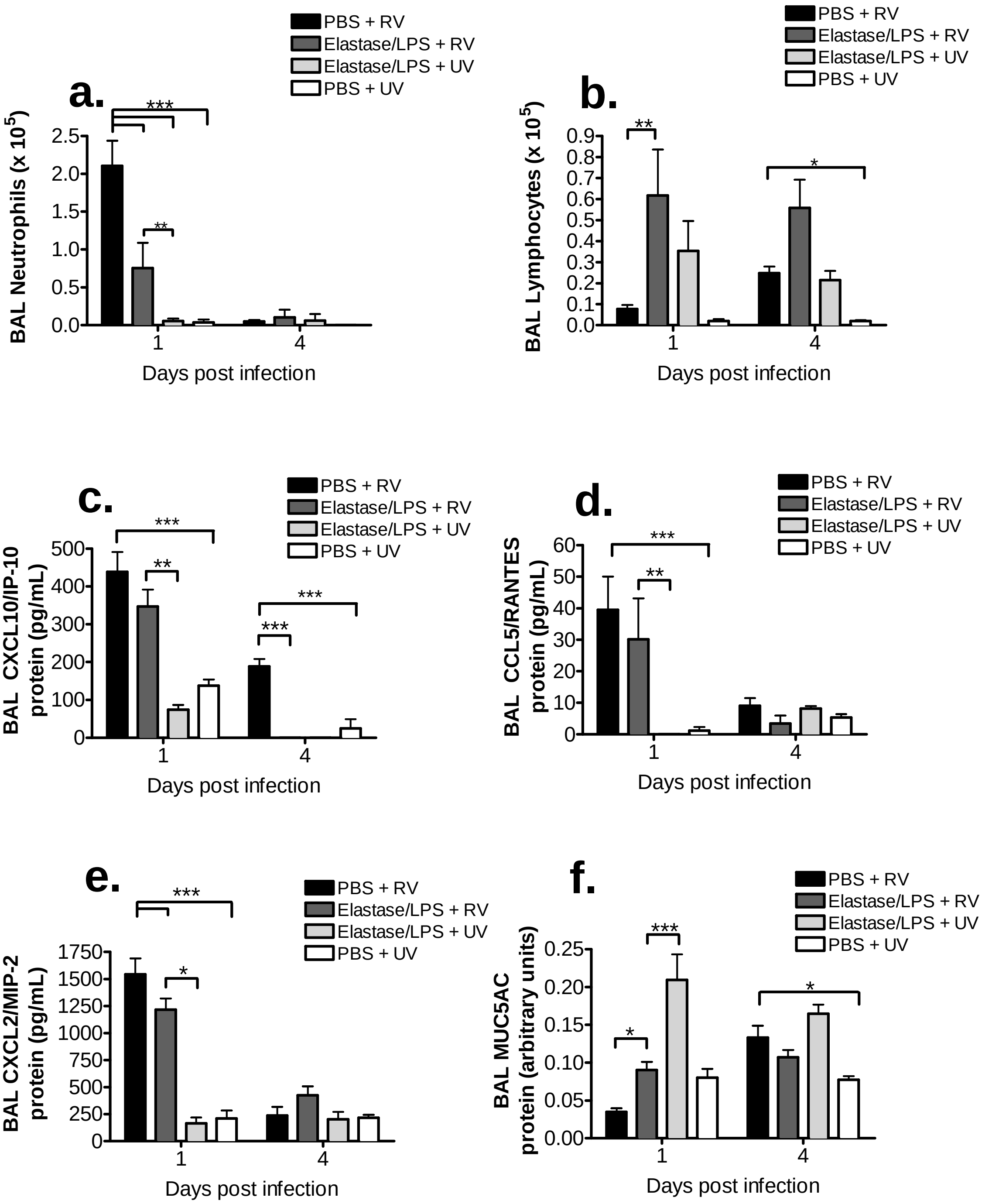

Figure S2: Effect of four dose elastase/LPS administration on cellular airways inflammation, inflammatory cytokines and mucin production in RV infected mice.

Mice were challenged intranasally with elastase on day 1 and LPS on day 4 of each week, or challenged with PBS control, for four weeks. At day 7 following final LPS or PBS challenge, mice were infected with rhinovirus 1B or UV-inactivated RV1B (UV). (a) Neutrophil and (b) lymphocyte numbers in BAL were enumerated by cytospin assay. (c) CXCL10/IP-10, (d) CCL5/RANTES, (e) CXCL2/MIP-2 and (f) MUC5AC protein in BAL was measured by ELISA n=5 mice/group. Data were analysed by two-way ANOVA and Bonferroni post-test. * $p<0.05$ **<0.01 ***$p<0.001$.
